# Efficacy of praziquantel on *Schistosoma haematobium* and re-infection rates among school-going children in the Ndumo area of uMkhanyakude district, KwaZulu-Natal, South Africa

**DOI:** 10.1186/s40249-017-0293-3

**Published:** 2017-04-07

**Authors:** Muhubiri Kabuyaya, Moses John Chimbari, Tawanda Manyangadze, Samson Mukaratirwa

**Affiliations:** 1 Discipline of Public Health Medicine, Howard College, University of KwaZulu-Natal, Durban, P.O Box, 4041 South Africa; 2 College of Health Sciences, University of KwaZulu-Natal, Durban, South Africa; 3 School of Life Sciences, University of KwaZulu-Natal, Durban, South Africa

**Keywords:** *Schistosoma haematobium*, Praziquantel efficacy, Reinfection, Incidence, Schoolchildren, Ndumo, KwaZulu-Natal, South Africa

## Abstract

**Background:**

Despite its low cure rates and possible resistance, praziquantel (PZQ) is the only drug available for schistosomiasis treatment. Hence, monitoring its efficacy is crucial. This study assessed the efficacy of PZQ, determined re-infection and incidence rates of *Schistosoma haematobium* infection among school-going children in the Ndumo area, KwaZulu-Natal.

**Methods:**

A cohort of 320 school-going children (10 – 15 years) in 10 primary schools was screened for *S. haematobium* infection using the filtration technique. Infected children were treated at different times and hence were divided into two sub-cohorts; A1 and A2. Non-infected children constituted the sub-cohort B. Children who continued excreting viable eggs 4 weeks post-treatment received a second dose of PZQ. Re-infection rates were determined in sub-cohort A1 and A2 at 28 and 20 weeks post-treatment, respectively. Cure rates (CR) and egg reduction rates (ERR) were calculated. Incidence rate was assessed 28 weeks post baseline survey using children that were negative for schistosome eggs at that survey. Analysis of data was done using the Chi square and the Wilcoxon rank test. A 95% confidence interval with a *P*-value < 0.05 determined significance.

**Results:**

At baseline, 120 (37.5%) of the 320 study participants were found infected with *Schistosoma haematobium*. Heavy infections accounted for 36.7%. The calculated cure rates were 88.07% and 82.92% for females and males, respectively. Egg Reduction Rates of 80% and 64% for females and males were observed 4 weeks after the initial treatment. After the second treatment, CR was 100% in females and 50% in males with an ERR of 100% in females and 70% in males. At 20 and 28 weeks post treatment, reinfection rates of 8.03% and 8.00% were observed, respectively, giving an overall rate of 8.1%. An incidence rate of 4.1% was observed 28 weeks after the baseline screening.

**Conclusions:**

The study indicated high CR while the ERR was low suggesting a reduced PZQ efficacy. The efficacy improved among females after the second dose. Re-infection rates at 20 and 28 weeks post-treatment were low. The study also indicated a low incidence rate for the 28 weeks period.

**Electronic supplementary material:**

The online version of this article (doi:10.1186/s40249-017-0293-3) contains supplementary material, which is available to authorized users.

## Multilingual abstracts

Please see Additional file 1 for translations of the abstract into the five official working languages of the United Nations.

## Background

Worldwide, urinary and intestinal schistosomiasis continue to be public health problems in tropical and sub-tropical areas [1]. An estimated 779 million people are exposed to the infection and the highest burden of the disease is in Africa, particularly the sub-Saharan region that accounts for roughly 90% of the infection [2]. Globally, in 2012 at least 249 million required preventive treatment but only 42.1 million were treated. Among those treated, 84.5% were in African countries [3, 4]. The number of treated people increased to 61.6 million in 2014, representing 20.7% of those requiring preventive treatment. Of the 49.2 million children treated, 43.3 million (83.4%) were in the African region [5]. The number of people needing annual treatment in South Africa was recently indicated to be 5.2 million [6].

In endemic areas, people contract *schistosome* infections during activities that bring them in contact with water infested with cercaeriae released by intermediate host snails [1, 4]. Supply of potable water, improvement of sanitation combined with preventive chemotherapy using praziquantel are considered the mainstay strategies to curb the burden of schistosomiasis [4]. Environmental concerns and high costs associated with control of intermediate host snails have hindered the achievement of a successful overall schistosomiasis control strategy [7]. Nevertheless, Brazil, Cambodia, Egypt, China and the Philippines have made satisfactory progress in reducing morbidity and mortality due to schistosomiasis [8].

To date, praziquantel administered at the standard oral single dose of 40 mg/kg body weight is the only drug recommended by WHO for preventive chemotherapy [1, 4]. Studies have indicated that it drastically reduces morbidity and transmission of schistosomiasis with a high cure rate (CR) and satisfactory egg reduction rate (ERR) [9, 10]. Few reports of treatment failures have been reported with praziquantel in endemic areas [10, 11].

Drug trial studies comparing the use of the recommended regimen of 40 mg/kg to 60 mg/kg single dose found that both regimens had comparable efficacy [12, 13] with the 60 mg/kg dose having a significantly higher rate of mild and transient side effects than the 40 mg/kg dose [12]. Hence, a dose of 60 mg/kg split in two equal doses has been suggested in treatment to prevent side effects and to kill immature worms [14].

Controversies about the use of a repeated dose within 2–8 weeks following the initial dose of praziquantel have arisen [15]. Notably, higher parasitological improvements were observed after a repeated second dose than it was after at the first dose; and the cure rate also differed between species, being higher for *S. mansoni* than for *S. haematobium* [15]. However, praziquantel is only active on adult worms but not on immature worms [16]. Thus, a combination of praziquantel with antimalarial drugs (artemether, artesunate) possessing anti-schistosome properties of killing immature worms has been suggested [16, 17].

Rapid assessment of incidence in a community is significantly sensitive for indicating periodic lapses in the quality control [18]. Despite the efficacy of praziquantel at the standard dose, rapid re-infections have been reported [19, 20]. Factors such as socio-demography [21], level of schistosomiasis prevalence [22], and seasonal variations [23] in the area have been reported to influence the re-infection and incidence rates.

Schistosomiasis is endemic in South Africa, particularly in the KwaZulu-Natal (KZN) province. The Department of Health in collaboration with the Department of education implemented a national helminth control program promoting regular treatment for schistosomiasis and soil transmitted helminths in all primary schools between 1997 and 2000 [23, 24]. uMkhanyakude is among districts with the a very high burden of schistosomiasis [25] in KZN province. Prevalence of 68% and 16.6% were reported in 1998 in the northernmost part and southernmost part of the district, respectively [23, 26]. A study conducted in 2011 in the Ugu district of KwaZulu-Natal reported that 44.3% of school going children were reached during a mass treatment campaign implemented by the provincial Department of Health [6].

Although prevalence and spatial distribution of schistosomiasis have been reported in the district, less emphasis has been given to the efficacy of praziquantel and the subsequent re-infection rates following treatment in uMkhanyakude district. Information on the efficacy of praziquantel and the infection rates may help in evaluating policies and strategies that guide schistosomiasis control activities in the district, particularly in the Ndumo area. Moreover, the existing data might need to be updated since the last study that assessed efficacy of PZQ in the area was about 18 years ago [23]. Thus, we investigated the efficacy of praziquantel, the re-infection and incidence rates among school-going children aged 10 – 15 years.

## Methods

### Study area and population

**Fig. 1:**
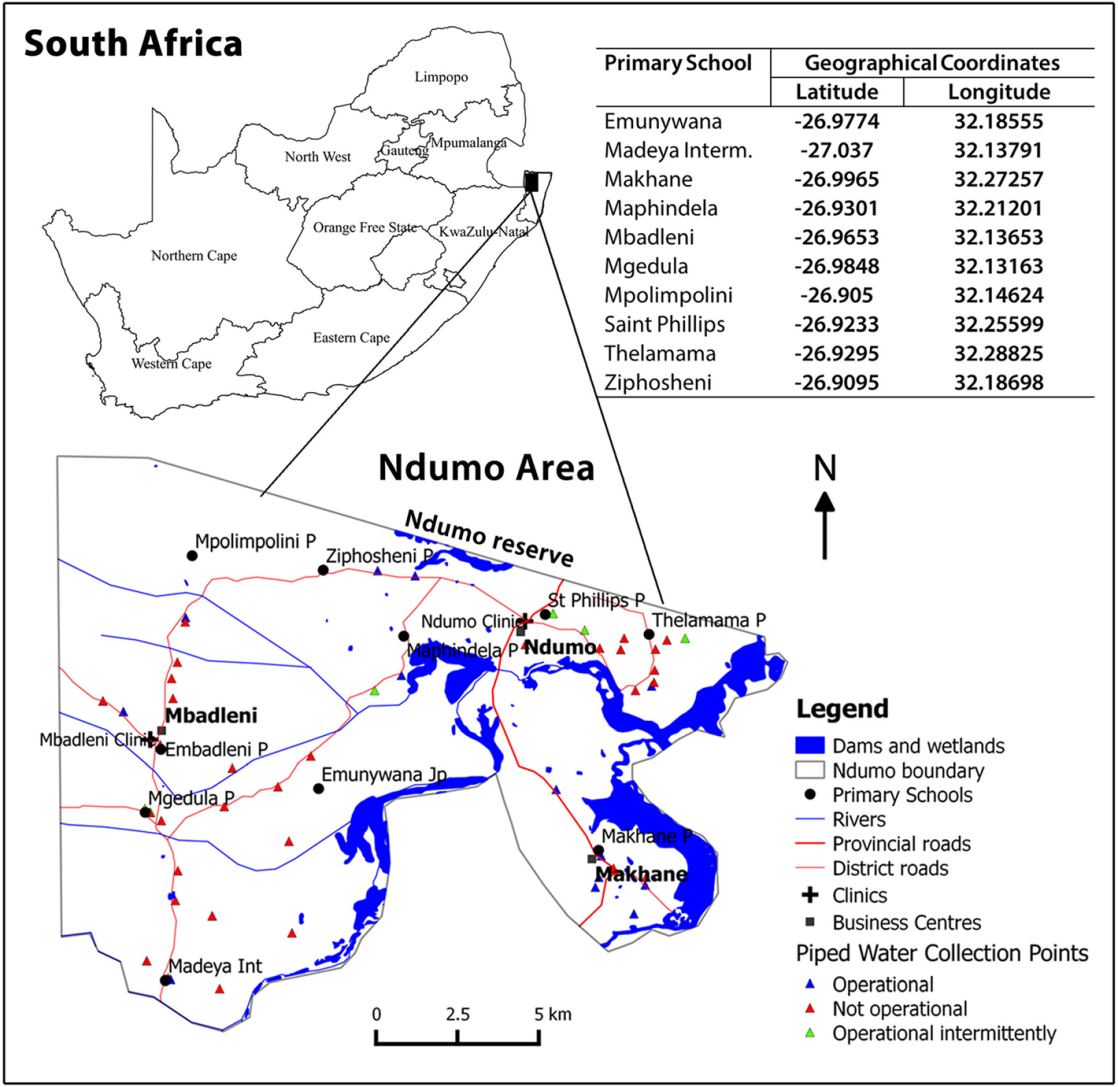
Map of Ndumo area and coordinates of surveyed schools, uMkhanyakude district, South Africa, adapted from Manyangadze et al. [28]

This study was conducted in Ndumo area located at the northernmost part of uMkhanyakude district in KwaZulu-Natal (KZN) province, South Africa (Fig. 1). The district extends over 12 818 km^2^ limited to the East by the Indian ocean, to the West by Zululand district, to the North by Mozambique, to the Northwest by Swaziland, and to the South by uThungulu district [27]. uMkhanyakude district is generally arid with a sub-tropical climate characterised by a hot and humid summer (November – February); and a cooler and drier winter (June – August). Its hydrologic network is constituted by rivers (e.g. mainly Ingwavuma and Pongola), streams, dams (e.g. Pongola) and ponds [25]. There is limited access to piped water [28]. Thus, people rely on open sources of water (river and dams) to sustain their daily domestic needs of water [27]. The unemployment rate is high; 46.18% in 2003 [27] and 53.00% in 2010 [25]. Moreover the district, particularly the Ndumo area is considered as the gateway from Mozambique to South Africa [29]. Thus imported cases of schistosomiasis are likely to come in through Mozambique, one of the countries with the highest burden of schistosomiasis worldwide [4]. uMkhanyakude district also has the highest prevalence of HIV infection and schistosomiasis infection in the country [25].

### Study design and sampling

**Fig. 2:**
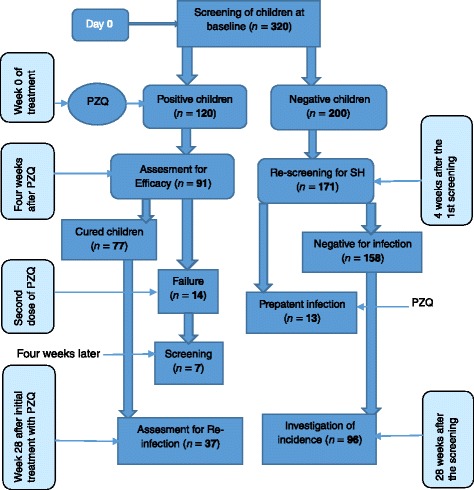
Schematic diagram of the study design

A prospective cohort of 10–15 years old school-going children whose parents signed consent forms and the children assented constituted the study sample. Study participants were randomly sampled from all primary schools located (Fig. 1) in the Ndumo area (10). They were initially screened for schistosomiasis at the end of June 2015 and follow up screening was done in early March 2016 (Fig. 2).

A sample size of 246 primary school-going children was determined using the formula as described by Lwanga (1991) and Daniel (1999) [30]:

$$ N=\frac{Z^2\mathrm{P}\left(1-\mathrm{P}\right)}{d^2} $$

Where *Z* statistic is 1.96 for the confidence level of 95%; *P* is 0.8 as the expected proportion of the characteristic to be measured in the study area [25]*; d* is the precision of 0.05 for 95% confidence interval.

Since it was a prospective study, attritions were expected for various reasons; unsigned consent and assent forms, absence of participants at school during the day of screening, failure to provide urine sample or not being available during subsequent surveys. We therefore multiplied *N* (246) by 2.5 to estimate the baseline sample size of the study at 615 of which only 320 children participated in the study. School registers were used to randomly select the study participants taking into account the sex ratio. Children that were screened for *S. haematobium* in the initial pre-treatment survey were divided into 2 cohorts A and B. Cohort A comprised of participants that were initially found positive for *S. haematobium* infection. They were treated and followed-up to determine praziquantel efficacy and re-infection. However, because of challenges associated with treatment logistics, cohort A was divided into two sub-cohorts. Sub-cohort A1 included children from five primary schools treated in July 2015 and sub-cohort A2 included children from the other five schools treated in September 2015. Thus, sub-cohort A1 was rescreened 28 weeks post treatment while sub-cohort A2 was rescreened after 20 weeks to determine the re-infection rate. Cohort B included school-going children that were initially negative and re-screened at the end of the study (28 weeks later) to determine incidence.

### Data collection

#### Parasitology survey

At each survey study participants were asked to provide 50 ml of urine in plastic containers between 10 AM and 2 PM, the likeliest time to get high load of eggs [31]. The urine samples were stored in wooden boxes with a lid under a shade to protect them from the sunlight until the time of processing and examination on the same day. Ten millilitres of urine were examined using the filtration technique for detection of *S. haematobium* eggs [31, 32].

Given the difference in the treatment period (8 weeks) which led to differences in the period of exposure of sub-cohort A1 and A2, re-infection cases were determined at week 28 post treatment in sub-cohort A1; and at week 20 post treatment in sub-cohort A2. Another parasitology survey was conducted to detect new cases of infection (incidence) in cohort B at 28 weeks from the baseline.

#### Treatment with Praziquantel

Treatments of infected children at the 10 primary schools was done by nurses based at local clinics of the Jozini municipality Department of Health with the assistance of the research team. The first and second treatments were done in July and September 2015 for sub-cohort A1, and in August and October 2015 for sub-cohort A2. Study participants who tested positive for *S. haematobium* were treated with praziquantel (biltricide, lot number: 364415) at the standard dose of 40 mg/kg body weight as recommended by the WHO [4]. The drug was administered orally after the child had eaten at least 4 slices of bread with a cup of fruit juice. To ensure adherence, every child took the tablets in front of the research team members. Children that continued excreting viable eggs in the urine 4 weeks after the initial treatment received further treatment with PZQ at the same dose of 40 mg/kg body weight. They were re-screened after 4 weeks and those that remained positive after the repeated treatment were referred to the clinics for follow up.

### Data management and statistical analysis

The SPSS 24 version was used for data analysis [33]. Positive *S. haematobium* infection was defined as a sample with number of eggs greater than zero in 10 ml of urine. The number of infected children with *S. haematobium* over the total number screened was defined as prevalence. Intensity of infection was classified as “light” for less than 50 eggs/10 ml and “heavy” for equal or more than 50 eggs/10 ml for *S. haematobium* [34]. As the assessment for PZQ efficacy followed the same process in both cohorts (A1 and A2) the results were pooled together for interpretation. Efficacy was defined as an absence of eggs in the specimen 4 weeks post treatment in participants who initially tested positive. To assess the praziquantel efficacy, cure rate (CR) and egg reduction rate (ERR), the following formulae used in previous studies [35, 36] were used.

Since data were not normally distributed even after log transformation, geometric means were calculated only for positive values (greater than 0). Wilcoxon signed ranks test was therefore used for comparison of geometric mean egg accounts.

Pearson Chi square test was performed to assess the association between the infection intensity with age and gender. It was also used to ascertain the association of the re-infection and incidence rates with the infection intensity, gender and the age. Participants that continued excreting viable eggs after they had received PZQ treatment were regarded as drug failure cases. Re-infection cases were defined as those that were positive for schistosomiasis on the initial examination and became negative on the second examination reverting to positive on the subsequent parasitology survey. Participants that became negative after the administration of the second dose of PZQ were excluded from the analysis.

Incidence was defined as the occurrence of new cases of infection among those who tested negative at the initial parasitology survey. Children that tested positive 4 weeks after the initial parasitology survey were considered as pre-patent cases and were excluded from the analysis. Considering the difference in the treatment period, re-infection rate was assessed at 20 weeks post treatment for cohort A1 at 28 weeks for cohort A2.

Children that had left school during the subsequent surveys or withdrew from the study or failed to provide urine specimens were excluded from the data analysis.

95% Confidence interval were used with *P* value < 0.05 as the statistical significance.

## Results

### Baseline parasitology

In the baseline parasitology survey, the mean age of participants was 12.9 years. Out of the 320 screened school-going children aged 10 – 15 years enrolled in the study, 120 (37.5%) tested positive for *S. haematobium* infection. 63 (60.8%) were females while 47 (39.2%) were males. 44 (36.7%) had heavy infections with females having the highest rate (54.5%).

13 and 14 years old participants had the highest rate of heavy infection (25.5% each) as shown in Table 1. However, the differences in intensity of infection between ages (*χ* ^2^ = 4.886, *P* = 0.430) and males and females (*χ* ^2^ = 1.153, *P* = 0.283) were not significant.

**Table 1:** *Schistosoma haematobium* infection intensity at baseline amongst primary school-going children in Ndumo area, uMkhanyakude district

Parameters		Infection intensity		*χ* ^2^	*P* value
		Light	Heavy		
Age	10 years	1 (1.3%)	1 (2.3%)	4.886	0.430
	11 years	20 (26.3%)	6 (13.6%)		
	12 years	14 (18.4%)	6 (13.6%)		
	13 years	19 (25.0%)	11 (25.0%)		
	14 years	11 (14.5%)	11 (25.0%)		
	15 years	11 (14.5%)	9 (20.0%)		
Gender	Female	49 (64.5%)	24 (54.5%)	1.153	0.283
	Male	27 (35.5%)	20 (45.5%)		

Light infection: < 50 eggs/10 ml, Heavy: ≥ 50 eggs/10 ml

### Treatment and efficacy of praziquantel

Of the 120 infected school going children treated with praziquantel, 29 were not at school during the follow-up survey representing an attrition rate of 24.16%. They were therefore, excluded from the analysis. Ninety-one participants were screened for infection four weeks after they had received the first dose of PZQ at 40 mg/Kg body weight (Table 2). Out of the 91 children, 77 (84.62%) were cured; 43 (55.84%) were females and 34 (44.16%) were males. However, 14 (15.38%) participants continued excreting viable eggs. CR of 86% and ERR of 80% were found in females while for males the same rates were 82.9% and 64%, respectively following administration of the first dose as shown in Table 2. Higher CR was observed amongst 11 years old children (86.6%) while failure rates were higher amongst 12, 13 and 14 years old participants accounting for 4 children (28.6%) in each group. All children that continued excreting eggs 14 (15.38%) had light infections. The arithmetic mean decreased in both females and males from 103.04 to 2.08 and 99.13 to 3.61, respectively. Of the 14 cases of treatment failure that received the second dose of PZQ, 7 (50%) were re-screened. The other 7 had left school, resulting in an attrition rate of 50.0%. The CR was 100% in females compared to 50% in males with a ERR of 100% in females and 78% (Table 2).

**Table 2:** Praziquantel efficacy (PZQ) against *Schistosoma haematobium* after the first and second treatment at 40 mg/kg

Gender	Baseline		4 weeks after the 1^st^ dose of PZQ						4 weeks after the 2^nd^ dose of PZQ					
	Positive	AM/10 ml	No assessed	No cured	Failure cases	CR	AM/10 ml	ERR	No Assessed	No cured	No of Failure	CR	AM/10 ml	ERR
Female	73	103.04	50	43	7	86.0	2.08	80	3	3	0	100	0	100
Male	47	99.13	41	34	7	82.9	3.61	64	4	2	2	50	22	78

*CR=* Cure Rate, *ERR*= Egg reduction rate, *AM/10 ml* Arithmetic Mean of egg counts per 10 ml urine

Comparison of the geometric mean of egg counts comparison post administration of PZQ gave a *Z* value of −1.572 with a *P* value of 0.116 between the baseline and after the first dose; and a *Z* value of −0.447 with a *P* value of 0.655 between the baseline and after the second dose administration. No statistical significance was found (*P* value > 0.05) between geometric means of eggs for the different periods of intervention for the efficacy assessment.

### Re-infection

Table 3 shows that 37 children were re-screened to determine the re-infection rate of which sub-cohort A1 accounted for 25 and sub-cohort A2 for 12. Of the 25 followed in sub-cohort A1, 23 (92.0%) participants remained negative while 2 (8.0%) became infected 28 weeks post treatment and only females were infected. In the sub-cohort A2, of the 12 screened children, 11 (91.7%) remained negative while only 1 (8.3%) child got re-infected. Overall, out of the 37 children that were cured for schistosomiasis, 34 (91.9%) remained negative and 3 (8.1%) contracted the infection after treatment. Eleven years old children had the highest cure rate while 12 years old were more affected but there was no statistical significant difference (*χ* ^2^ = 7.600, *P* = 0.107). Two female participants were re-infected (66.7%) and this was among those that had light infection at the baseline.

**Table 3:** Occurrence of re-infection cases of *Schistosoma haematobium* post-treatment amongst school-going children in Ndumo area, KwaZulu-Natal

Cohort	Gender	Baseline	4 weeks post treatment				Reinfection assessment		
		Positive children	No. of Screened	No. of attritions (%)	Cured children (%)	No. of Failure (%)	Screened children	Negative (%)	Reinfection Cases (%)
A1 (28 weeks)	Female	49	36	13 (26.5)	32 (88.9)	4 (11.1)	17	15 (88.2)	2 (11.7)
	Male	28	26	2 (7.1)	22 (84.6)	4 (15.3)	8	8 (100.0)	0 (0.0)
A2 (20 weeks)	Female	24	14	10 (41.6)	11 (78.57)	3 (21.43)	5	5 (100.0)	0 (0.0)
	Male	19	15	4 (21.0)	12 (80.0)	3 (20.0)	7	6 (85.7)	1 (14.3)
	Total	120	91	29 (24.1)	77 (84.6)	14 (15.4)	37	34 (91.9)	3 (8.1%)

### Incidence

At the baseline parasitology survey, 200 children tested negative of which 171 participated in the follow up survey. Hundred and eight were females and 63 were males Of the 96 cases used to determine incidence, 92 (95.83%) remained negative for *S. haematobium* infection while 4 (4.17%) were new infections of which 3 (75%) were females (Table 4). Fifty percent of the new infections were among 11 years old children but there was no significant difference between the age (*χ* ^2^ = 3.230, *P* = 0.664).

**Table 4:** Occurrence of new-infections of *Schistosoma haematobium* at 28 weeks amongst children in the Ndumo area

Gender	Baseline				Incidence assessment at 28 weeks			
	1^st^ Screening	2^nd^ Screening	Prepatent Infection	Negative	Screened	Attrition Rate	Negative	New Infection
Female	126	108	7 (6.4)	101 (93.6)	62	39 (36.1)	59 (95.2)	3 (4.8)
Male	74	63	6 (9.5)	57 (90.5)	34	25 (39.7)	33 (95.0)	1 (3.0)
Total	200	171	13 (7.6)	158 (92.4)	96	64 (37.4)	92 (95.9)	4 (4.1)

## Discussion

At baseline survey, *Schistosoma haematobium* infection in the study area was relatively low (37.5%) compared to 68.8% prevalence reported 18 years ago [23]. The drastic reduction of the prevalence was probably because of a treatment programme targeting school-going children in primary schools that has been implemented for 3 years in the study area [24]. Our findings suggest that administration of praziquantel once every 2 years according to the WHO recommendation [35] is appropriate for Ndumo area. Females were more affected than males and more likely to have contact with contaminated water than males since fetching water and washing clothes are considered as female duties in the study area [37]. Thirteen years and 14 years old children had the highest rate of heavy infection that decreased with age. The findings are consistent with those found in a similar study in Ethiopia [38]. This may be attributed to intense water contact activities that adolescents are usually involved in but decreases with the age. Moreover, the study reported heavier infections in females than in males. Our results corroborate with those reported in a survey conducted in Nigeria [39]. The fact that in rural communities, females are more exposed to water contact activities (fetching water from river/dam, washing clothes and dishes) than males may explain these observations. However, in other studies [38, 40] heavy infections were predominantly recorded among males. This, however depends on socio-environmental factors where the studies were conducted. For instance, a fishing community where mainly men do the fishing might have more males infected while in a community with market gardening more women might be infected.

The CR and ERR in both females and males 4 weeks post treatment suggested that the efficacy of praziquantel after the first single standard dose was reduced. Similar results were reported in studies conducted in rural communities of Abeokuta of Nigeria [36], in Senegal [41], and in Loum, Cameroun [42]. In contrast to our findings, a study carried out in Zimbabwe found satisfactory efficacy with praziquantel after the first dose [43]. This might be because immature worms at first treatment were not killed and reached their maturity stage after treatment had been done. Thus, new mature worms would have produced viable eggs after the treatment. We found high treatment failures amongst 13 and 14 years old children with heavy intensity. CR and failure cases observed were consistent with findings in a study conducted in Abeokuta, Nigeria [36]. A high load of eggs in the urine indicates implicitly a high number of mature worms, which may require a higher dose than the standard dose. After the administration of the second treatment with PZQ, females were completely cured while CR remained low with ERR reduced in males. There was a significant improvement in CR and ERR in females compared to the outcome of the first dose. The efficacy of PZQ remained low in males. This is contradicts findings observed in others studies [36, 41] where the CR and ERR were higher after the administration of a second standard dose of PZQ than it was after the first round. Parameters such as infection intensity [36], low drug absorption, and high level of catabolism rather than the resistance of parasite have been associated with low efficacy of PZQ in endemic regions [43]. The findings in this study showed no significant difference in geometric mean egg counts at the different periods of treatment. This might have been due to light intensity of the infection after the administration of the first dose.

During the follow-up of children that were cured, a low re-infection rate was found (8.1%) 28 weeks post treatment. The persistent drought throughout the study period might explain our observations. Manyangadze et al. [43] showed that a considerable number of the transmission hotspots dried up during the study period, thus limiting the exposure of children to water contact. However, in comparison to a study conducted in the area 18 years ago, re-infection cases were observed only 41 weeks post treatment and seemed to be limited to the hot and rainy summer [23]. In contrast to our findings, other studies observed a rapid and high re-infection rate a few weeks following treatment [44]. In this case many factors have been incriminated in the occurrence of high re-infection rates such as ecological and seasonal factors [42], and being an area with high intensity infection [22]. People might get re-infected when they revert to their previous daily activities involving contact with water infested with snail intermediate hots after a successful treatment with praziquantel.

## Conclusions

The study showed a high CR and low ERR suggesting low efficacy of PZQ which improved in females after the second dose. An overall re-infection rate of 8.1% at 20 and 28 weeks post treatment was observed in the study during a drought period. The study also indicated an incidence rate of 4.1% over a period of 28 weeks. Since the study area is a moderate zone of transmission without any specific current control program, treatment with PZQ once every 2 years may keep the infection at low level of transmission. Moreover, while waiting for a more efficacious drug than PZQ to come on the market, control programs may consider the use of the repeated standard dose.

## Additional files

Additional file 1: Multilingual abstracts in the six official working languages of the United Nations. (PDF 705 kb)

## Abbreviations

AM: ﻿Arithmetic ﻿mean
CR: Cure rate
ERR: Egg reduction rate
GM: Geometric mean
KZN: KwaZulu-Natal
PZQ: Praziquantel
WHO: World Health Organisation
