# Supplementary material for: Efficacy of praziquantel on Schistosoma haematobium and re-infection rates among school-going children in the Ndumo area of uMkhanyakude district, KwaZulu-Natal, South Africa
Source: Infect Dis Poverty. 2017 Apr 7;6:83. doi: 10.1186/s40249-017-0293-3 (PMC5383960; doi:10.1186/s40249-017-0293-3)
Supplement: Supplementary file 1 — Multilingual abstracts in the six official working languages of the United Nations. (PDF 705 kb) [file 40249_2017_293_MOESM1_ESM.pdf]

Translation of the abstract into the five official working languages of the United Nations

فعالية برازيكوانتيل على البلهارسيا الدموية وعلى معدلات عودة العدوى بين الأطفال في سن المدرسة في منطقة ندومو التابعة لمقاطعة يومخاياكودي، كوازولو ناتال، جنوب أفريقيا

موهوبيري كابويايا، موسيس جون تشيمباري، تاواندا مانيانغادزي، سمسون موكاراتيروا–

الملخص

الخلفية: على الرغم من انخفاض معدلات الشفاء وإمكانية حدوث المقاومة، فإن برازيكوانتيل (PZQ)هو الدواء الوحيد المتاح لعلاج البلهارسيا. وهكذا، فإن مراقبة فعاليته أمر بالغ الأهمية. قيّمت هذه الدراسة فعالية PZQ، حددت معدلات إعادة العدوى والإصابة بعدوى البلهارسيا الدموية بين الأطفال في سن المدرسة في منطقة ندومو، كوازولو ناتال.

الطرق: أجري مسح على مجموعة من 320 طفلا في سن المدرسة (10 – 15 سنة) من 10 مدارس ابتدائية للكشف عن وجود عدوى البلهارسيا الدموية باستعمال تقنية الترشيح. عولج الأطفال المصابون في أوقات مختلفة، وبالتالي قسموا إلى قسمين فرعيين؛ A1وA2 . شكّل الأطفال غير المصابين الجماعة الفرعية B. الأطفال الذين واصلوا إطراح البيوض القابلة للحياة بعد 4 أسابيع من المعالجة تلقوا جرعة ثانية منPZQ . حدّدت معدلات عودة العدوى في المجموعات الفرعية A1 و A2 بـ 28 و 20 أسبوعا بعد العلاج، على التوالي .تم حساب معدلات الشفاء (CR) ومعدلات انخفاض إطراح البيوض(ERR) . قُيّم معدل الإصابة بعد 28 أسبوعا من المسح الأساسي بالاستعانة بالأطفال الذين كانوا سلبيين لبيوض البلهارسيا في ذلك الاستقصاء. وقد تم تحليل البيانات باستخدام $\chi 2$ واختبار رتبة ولكوكسُن. وكان فاصل الثقة البالغ 95٪ مع P>0.05 هما محددا الأهمية.

النتائج: عند الخط القاعدي، كان 120 (37.5٪) من بين الـ 320 طفلا مشاركا في الدراسة مصابين بعدوى البلهارسيا الدموية. وشكلت العدوى الشديدة ما نسبته 36.70٪ من الحالات. كانت معدلات الشفاء 88,07% و 82,92% عند الإناث والذكور، على التوالي. وقد لوحظ انخفاض معدل البيوض بمقدار 80٪ و 64٪ للإناث والذكور بعد 4 أسابيع من العلاج الأولي . بعد المعالجة الثانية، كان معدل الشفاء 100% عند الإناث و 50% عند الذكور وبلغ انخفاض معدل البيوض 100% عند الإناث و 70% عند الذكور. في الأسبوع 20 و الأسبوع 28 بعد العلاج، لوحظت عودة العدوى بمعدلات قدرها 8.03% و 8.00% على التوالي، وهو ما يجعل المعدل العام 8.10%. وقد لوحظ معدل إصابة قدره 4.10% بعد 28 أسبوعا من مسح الخط القاعدي.

الاستنتاج: أشارت الدراسة إلى معدلات مرتفعة لعودة العدوى بينما كان انخفاض إطراح البيوض منخفضا مما يشير إلى تناقص فعالية برازيكوانتيل. تحسنت الفعالية بين الإناث بعد الجرعة الثانية. كانت معدلات عودة الإصابة في الأسبوع 20 والأسبوع 28 بعد المعالجة منخفضة. و أشارت الدراسة أيضا إلى انخفاض معدل الإصابة لفترة الـ 28 أسبوعا.

Translated from English version into Arabic by Lina, through

TRANSLATORS
WITHOUT BORDERS

吡喹酮对埃及血吸虫病的疗效和南非夸祖鲁 - 纳塔尔省 Mkhanyakude 区 Ndumo 学龄儿童的重复感染率

Muhubiri Kabuyaya, Moses John Chimbari, Tawanda Manyangadze, Samson Mukaratirwa

摘要

引言：吡喹酮（PZQ）唯一治疗血吸虫病的药物，尽管它治愈率较低，也可能变得不敏感。因此，监测其疗效至关重要。本研究旨在评估 PZQ 的疗效，确定夸祖鲁 - 纳塔尔 Ndumo 地区学龄儿童的埃及血吸虫感染的发病率和重复感染率。

方法：使用过滤技术对 10 所小学中 320 名学龄儿童（10-15 岁）队列进行了埃及血吸虫感染的筛选。根据被感染儿童的治疗时期分为两个子队列：A1 和 A2。未感染儿童组成子队列 B。

PZQ 治疗后 4 周继续排出活卵的儿童接受第二次 PZQ 治疗。A1 和 A2 中分别在治疗后 28 和 20 周检测再感染率，计算治愈率（CR）和虫卵下降率（ERR）。进行基线调查确定血吸虫虫卵阴性的儿童，28 周后评估其发病率。运用卡方和 Wilcoxon 秩检验进行数据分析。运用 95%置信区间（*P* 值小于 0.05）确定是否有统计学意义。
结果：在基线调查中，发现 120 例学龄儿童（120/320, 37.5%）感染了埃及血吸虫。重度感染者占 36.70%。经计算，男童和女童的治愈率分别为 88.07%和 82.92%。在初诊后 4 周，女童和男童的虫卵减少率分别为 80%和 64%。第二次治疗后，女童的 CR 为 100%，男童的 CR 为 50%，女童的 ERR 为 100%，男童的 ERR 为 70%。在治疗后 20 和 28 周，观察到再感染率分别为 8.03%和 8.00%，总体再感染率为 8.10%。在基线筛选 28 周后观察到发病率为 4.10%。
结论：研究结果显示高 CR 低 ERR，表明 PZQ 疗效降低。在第二剂量后，对女童的功效改善。在治疗后 20 和 28 周的重复感染率低。该研究还表明 28 周时期的发病率较低。

Translated from English version into Chinese by Men-Bao Qian, through

CHINESE CENTER FOR DISEASE CONTROL AND PREVENTION
NATIONAL INSTITUTE OF PARASITIC DISEASES

De l'efficacité du praziquantel sur le *Schistosoma haematobium* et les taux de réinfection chez les enfants scolarisés dans la région Ndumo du district Umkhanyakude, KwaZulu-Natal, Afrique du Sud

Muhubiri Kabuyaya, Moses John Chimbari, Tawanda Manyangadze, Samson Mukaratirwa

Résumé
Contexte : Malgré ses faibles taux de guérison et une résistance possible, le praziquantel (PZQ) est le seul médicament disponible pour le traitement de la schistosomiase. Le suivi de son efficacité est donc crucial. Cette étude évalue l'efficacité du PZQ et détermine les taux de réinfection et d'incidence de l'infection *Schistosoma haematobium* chez les enfants scolarisés dans la région Ndumo, KwaZulu-Natal.
Méthodes : Un groupe de 320 enfants scolarisés (10-15 ans) dans 10 écoles primaires subit un dépistage de l'infection *S. haematobium* en utilisant la technique de filtration. Les enfants infectés sont traités à des moments différents, et sont pour ce faire divisés en deux sous-groupes ; A1 et A2. Les enfants non-infectés constituent le sous-groupe B. Les enfants qui continuent à excréter des œufs viables quatre semaines après le traitement se voient administrer une deuxième dose de PZQ. Les taux de réinfection sont déterminés au cours des 28 semaines suivant le traitement pour le sous-groupe A1, et au cours des 20 semaines suivantes pour le sous-groupe A2. Les taux de guérison (TG) et les taux de réduction des œufs (TRO) sont ensuite calculés. Le taux d'incidence est évalué 28 semaines après l'enquête de base, effectuée auprès d'enfants dont les résultats étaient négatifs pour les œufs de schistosomes. L'analyse des données est effectuée en utilisant le test du Khi carré et le test de Wilcoxon. Un intervalle de confiance de 95% avec un P <0,05 caractérise la fiabilité des résultats.
Résultats : Au départ, 120 des 320 participants à l'étude (37,5 %) se sont avérés positifs à l'infection du *Schistosoma haematobium*. Les infections graves représentaient alors 36,70 % des cas. Les taux de guérison calculés étaient de 88,07 % pour les femmes et de 82,92 % pour les hommes. Des taux

de réduction d'œufs de 80 % chez les femmes et de 64 % chez les hommes ont été observés quatre semaines après le traitement initial. Après le deuxième traitement, le TG est de 100 % chez les femmes et de 50 % chez les hommes, avec un TRO de 100 % chez les femmes et de 70 % chez les hommes. Des taux de réinfection de 8,03 % sont observés 20 semaines après le traitement, et de 8,00 % après 28 semaines de traitement, donnant ainsi un taux global de réinfection de 8,10 %. Un taux d'incidence de 4,10 % est observé 28 semaines après le dépistage de base.
Conclusion : L'étude révèle un TG élevé mais un TRO faible, suggérant une efficacité réduite du PZQ. L'efficacité s'est améliorée chez les femmes après la deuxième dose. Les taux de réinfection 20 et 28 semaines après le traitement sont faibles. L'étude a également indiqué un faible taux d'incidence au cours des 28 semaines du traitement.

Translated from English version into French by Laurence Masing, through

TRANSLATORS
WITHOUT BORDERS

Эффективность использования празиквантела при лечении *Schistosoma haematobium* и его воздействие на уровне повторного инфицирования среди школьников, проживающих в местности Ндумо, район Умханьякуде, провинция Квазулу-Натал, ЮАР

Мухубири Кабуяя, Моисей Джон Чимбари, Таванда Маньянгадзе, Самсон Мукаратирва

Аннотация

Базовая проблематика. Несмотря на низкий уровень излечения и возможного сопротивления, празиквантел (PZQ) является единственным доступным лекарственным средством для лечения шистосомоза. Таким образом, мониторинг его эффективности имеет решающее значение. В данном исследовании оценивалась эффективность PZQ, определялись уровни повторного инфицирования и заболеваемости *Schistosoma haematobium* среди школьников, проживающих в местности Ндумо, провинция Квазулу-Натал.
Методика. Группа, состоящая из 320 детей школьного возраста (10-15 лет) из 10 начальных школ, была проверена на инфицрование *S. haematobium* с помощью технологии фильтрации. Инфицированные дети лечились в разное время и, следовательно, были разделены на две подгруппы: A1 и A2. Неинфицированных детей составляли подгруппу B. Дети, которые продолжали выделять жизнеспособные яйца через 4 недели после лечения, получили вторую дозу PZQ. Показатели повторного инфицирования были определены в подгруппах A1 и A2 на 28-й и 20-й неделях после лечения, соответственно. Были рассчитаны показатели эффективности лечения (CR) и темпы сокращения числа яиц (ERR). Уровень заболеваемости оценивался 28 недель спустя после базового обследования детей, результат анализа на шистосомы у которых изначально был отрицательным. Анализ данных был проведен с использованием хи-квадрата и рангового критерия Уилкоксона. 95% доверительный интервал при $P < 0,05$ обусловил значимость исследования.

Результаты. На базовом уровне у 120 (37,5%) из 320 участников исследования была обнаружена инфекция *Schistosoma haematobium*. Тяжелые инфекции составили 36,70%. Рассчитанные показатели эффективности лечения были равны 88,07% и 82,92% для женщин и мужчин соответственно. У женщин и мужчин были зафиксированы темпы снижения числа яиц 80% и 64% соответственно через 4 недели после начала лечения. После второго курса лечения показатель эффективности лечения составил 100% у женщин и 50% у мужчин при темпе снижения числа яиц, равном 100% у женщин и 70% у мужчин соответственно. На 20-й и 28-й неделях после начала лечения показатели повторного инфицирования составили 8,03% и 8,00% соответственно, при общем показателе, равном 8,10%. Показатель заболеваемости 4,10% наблюдался через 28 недель после базового скрининга.
Вывод. Согласно результатам исследования, был зафиксирован высокий показатель эффективности лечения, в то время как темп снижения числа яиц был низким, что говорит о пониженной эффективности PZQ. Эффективность улучшилась у женщин после второй дозы. Показатели повторного инфицирования на 20-й и 28-й неделях после начала лечения были низкими. В ходе исследования также была зафиксирован низкий показатель заболеваемости в течение 28-недельного периода.

Translated from English version into Russian by Irina Zayonchkovskaya, through

TRANSLATORS WITHOUT BORDERS

Eficacia del praziquantel sobre el *Schistosoma haematobium* y tasas de reinfección entre los escolares de la zona de Ndumo, en el distrito de Umkhanyakude, KwaZulu-Natal, Sudáfrica

Muhubiri Kabuyaya, Moses John Chimbari, Tawanda Manyangadze, Samson Mukaratirwa

Resumen
Antecedentes: A pesar de sus bajas tasas de curación y una posible resistencia, el Praziquantel (PZQ) es el único medicamento disponible para el tratamiento de la esquistosomiasis. Por lo tanto, el seguimiento de su eficacia es crucial. Este estudio evaluó la eficacia del PZQ, determinó la reinfección y las tasas de incidencia de infección por *Schistosoma haematobium* entre los escolares en la zona de Ndumo, KwaZulu-Natal.
<Métodos: Se seleccionó una cohorte de 320 niños en edad escolar (10-15 años) en 10 escuelas primarias para detectar la infección por *S. haematobium* utilizando la técnica de filtración. Los niños infectados fueron tratados en diferentes momentos y por lo tanto se dividieron en dos sub-cohortes; A1 y A2. Los niños no infectados constituyeron la sub-cohorte B. Los niños que continuaron excretando huevos viables 4 semanas después del tratamiento recibieron una segunda dosis de PZQ. las tasas de reinfección se determinaron en las sub-cohortes A1 y A2 a las 28 y 20 semanas después del tratamiento respectivamente. Se calcularon las tasas de curación (CR) y de reducción de huevos (ERR). La tasa de incidencia se evaluó 28 semanas después de la encuesta inicial con niños que no tenían huevos de esquistosoma en esa encuesta. El análisis de los datos se realizó utilizando el Chi

cuadrado y la prueba de rango de Wilcoxon. Un intervalo de confianza del 95% con una *P* <0,05 determinaron la significancia.
Resultados: Al inicio del estudio, 120 (37,5%) de los 320 participantes en el estudio se encontraron infectados con *Schistosoma haematobium*. Las infecciones graves representaron el 36,70%. Las tasas de curación fueron 88,07% y 82,92% para mujeres y hombres, respectivamente. Se observó una tasa de reducción de los huevos de 80% y 64% para mujeres y hombres 4 semanas después del tratamiento inicial. Después del segundo tratamiento, la CR fue del 100% en las mujeres y el 50% en los hombres con una EER del 100% en las mujeres y el 70% en los hombres. A las 20 y 28 semanas post-tratamiento, se observaron tasas de reinfección de 8,03% y 8,00% respectivamente, con una tasa global de 8,10%. Se observó una tasa de incidencia de 4,10% 28 semanas después de la prueba inicial.
Conclusión: El estudio indicó una alta CR, pero una baja ERR, lo que sugiere una eficacia reducida del PZQ. La eficacia mejoró entre las mujeres después de la segunda dosis. Las tasas de reinfección 20 y 28 semanas después del tratamiento fueron bajas. El estudio también indicó una baja tasa de incidencia para el período de 28 semanas.

Translated from English version into Spanish by Amparo Muñoz, through

TRANSLATORS
WITHOUT BORDERS
